# Patient Shielding in Ultra-High-Resolution Cone-Beam CT of the Upper Extremity with a Twin Robotic X-Ray System

**DOI:** 10.3390/diagnostics15050596

**Published:** 2025-02-28

**Authors:** Andreas Steven Kunz, Stefan Weick, Jan-Peter Grunz, Andre Toussaint, Gary Razinskas, Anne Richter, Sonja Wegener, Andrea Wittig-Sauerwein, Thorsten Alexander Bley, Henner Huflage

**Affiliations:** 1 Department of Diagnostic and Interventional Radiology, University Hospital Würzburg, Oberdürrbacher Straße 6, 97080 Würzburg, Germany; 2 Department of Radiation Therapy and Radiation Oncology, University Hospital Würzburg, Josef-Schneider-Str. 11, 97080 Würzburg, Germany; 3 Department of Radiology, University of Wisconsin—Madison, 600 Highland Ave, Madison, WI 53792, USA

**Keywords:** radiation dose, cone-beam CT, patient shielding, shield protection, phantom study

## Abstract

**Background/Objectives:** Gantry-free cone-beam CT (CBCT) allows for ultra-high-resolution (UHR) upper extremity imaging in a comfortable tableside position. The aim of this study was to assess the organ-specific radiation burden and the effect of dedicated lead shielding in the UHR-CBCT of the wrist and elbow. **Methods:** A modified Alderson-Rando phantom was scanned with the tableside UHR-CBCT mode of a twin robotic X-ray system employing identical scan parameters for wrist and elbow imaging. An ion chamber was used in conjunction with an electrometer to obtain representative organ dose measurements for the eye lens, thyroid gland, breast tissue, and abdomen. All measurements were performed with and without lead shielding. **Results:** Irrespective of the examined upper extremity joint, the highest absorbed dose among the assessed organs was determined for the eye lens (wrist imaging: 0.10 ± 0.01 mGy, elbow imaging: 0.12 ± 0.01 mGy). The most effective organ dose reduction by means of shielding in wrist CBCT was achieved for the thyroid gland (−17%). In elbow CBCT, the abdomen (−48%) and the ipsilateral breast (−39%) benefited particularly from shield protection. **Conclusions:** Although shielding was more effective in elbow than wrist scans, the overall impact in terms of absolute dose reduction was marginal.

## 1. Introduction

Cone-beam computed tomography (CBCT) has emerged as an invaluable modality for musculoskeletal imaging tasks in recent years [1]. Particularly, in the intricate anatomical domains of the wrist and elbow, CBCT offers advantages due to its flat-panel detector design, boosting ultra-high spatial resolution courtesy of miniscule isotropic pixels, while simultaneously operating with low radiation exposure [2].

The diversity in CBCT systems is evident not only in their unique geometries. While some scanners employ a closed gantry, which leads to enhanced radiation containment, novel robotic scanners utilize a multipurpose approach with rotating arms that can be independently positioned for static or dynamic 2D imaging and also moved parallelly for acquisition of 3D projection data that can be reconstructed similarly to conventional multislice CT (MSCT) [3]. In a gantry-based approach, patients need to be positioned in the so-called superman stance, i.e., in a prone position with one arm outstretched above the head level. However, in the context of acute injury or recent surgery, individuals are often unable to adopt or maintain the superman position, hence requiring the affected limb to be placed adjacent to or on top of the radiation-sensitive body trunk, resulting in hampered image quality and severely increased dose burden. Conversely, gantry-free imaging allows for positioning of the patient in a more comfortable tableside position, facilitating high-resolution scans with reduced radiation dose.

While radiation protection efforts have intensified considerably in recent years, the use of ancillary patient shielding in extremity CBCT has not been thoroughly investigated. As previously demonstrated for MSCT scanners, strategical placement of lead shields over organs outside of the intended scan area can effectively mitigate the scatter radiation that might otherwise contribute to unnecessary dose exposure of healthy tissues [4]. Shielding techniques are particularly relevant in scenarios where scatter radiation can affect radiation-sensitive tissues, such as the thyroid gland or reproductive organs [5,6,7]. In conventional gantry-based MSCT, for example, various societies advocate for specific protection of the thyroid gland when performing head scans [8].

Whether shielding is also relevant in gantry-free CBCT remains to be investigated, since no trial has focused on this aspect to date, regardless of the anatomical region. To address this research gap for two of the most common imaging tasks, the presented study analyzes not only the organ-specific radiation burden but also the effect of dedicated patient shielding on radiosensitive tissues adjacent to the irradiated area in wrist and elbow CBCT.

## 2. Material and Methods

### 2.1. Study Setup

CBCT imaging was performed with a multipurpose twin robotic X-ray system (Multitom Rax, Siemens Healthineers, Forchheim, Germany). This scanner is equipped with two ceiling-mounted telescopic arms moving along predefined trajectories with an asymmetrical source-to-detector distance of 115 cm and a sweep angle of 200 degrees. One of the arms holds the X-ray tube, which can generate currents between 0.5 and 800 mAs and voltages between 40 and 150 kV. In accordance with IEC guidelines, the focal spot size of the X-ray tube measures 0.6 mm. The second arm is equipped with a flat-panel detector, which has an input field of 213 mm × 213 mm and a 3D matrix of 1440 × 1440 pixels with an effective size of 149 µm. A total of 318 projection images are recorded during each examination. In clinical routine, images are reconstructed with using a scanner-side workstation with dedicated software (syngo.via View&GO, Siemens Healthineers). A sharp bone kernel, which is characterized by spatial frequencies of 16.7 line pairs per cm at 50% and 25.4 line pairs per cm at 10% of the modulation transfer function, is used for three-planar reformatting. In addition to axial, coronal, and sagittal reconstructions with 1 mm slice thickness, a second stack of axial images is reconstructed with 0.5 mm slice thickness for PACS-based free-hand reformatting [2]. No extra sharpening or denoising algorithms are employed during post-processing. For optimal visualization of bone tissue, the default window width/level are 3000/1000 Hounsfield units. These presets can be altered for soft tissue evaluation, though.

**Figure 1:**
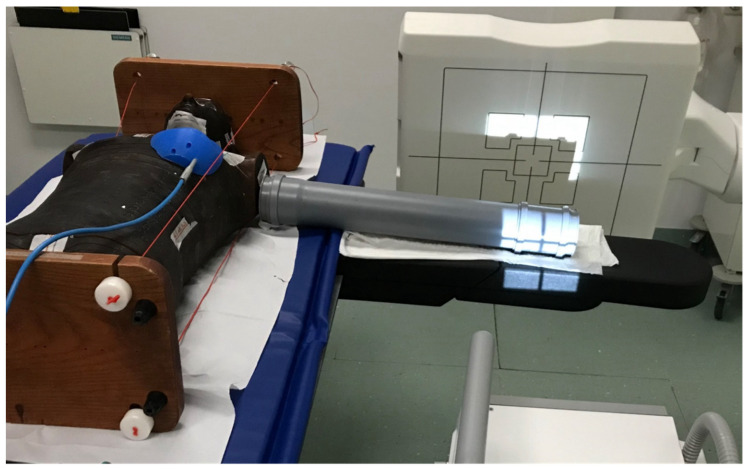
Depiction of the modified Alderson-Rando phantom that was examined with the tableside cone-beam CT scan mode of a multipurpose twin robotic X-ray system.

In the presented study, dose measurements were performed using an anthropomorphic Alderson-Rando phantom. A water-filled plastic tube of 43 cm length and 9 cm diameter was added to the phantom at shoulder height to simulate scatter conditions comparable to a left arm (Figure 1). Either the distal part of the tube (wrist) or the central part of the tube (elbow) was imaged. A clinically established high-quality scan protocol in ultra-high-resolution scan mode was employed with identical settings for wrist and elbow imaging. Detailed scan parameters are listed in Table 1. The high-quality protocol was chosen to reduce measurement uncertainties owing to the higher overall detector signal. The output ratio as indicated by the dose-area product was verified by measurements in the direct beam. All measurements were performed in the tableside position similar to clinical routine imaging, where the affected arm was positioned in 90° lateral abduction and the wrist is placed in pronation position. Institutional review board approval was not required for this experimental phantom study, since this phantom-based investigation did neither involve humans nor animals.

### 2.2. Radiation Dosimetry

Dose-area products were obtained from the automatically created scan report for all examinations. Additionally, a Farmer 30013 ion chamber (PTW Dosimetry, Freiburg, Germany) was used in conjunction with a Unidos electrometer (PTW Dosimetry) to obtain dose measurements at different positions within or on top of the anthropomorphic phantom. The Semiflex 31010 chamber had previously been calibrated in terms of dose-to-water by a secondary standard calibration laboratory for X-ray energies at similar conditions (depth, field size, and beam quality). Background radiation was measured prior to data acquisition and subtracted from the electrometer readings. To achieve this, the electrometer was zeroed before measurements and background radiation was collected for 75 s before automatic subtraction.

For obtaining the lens dose, the ion chamber was placed on top of the left eye of the phantom without any additional build-up. For the thyroid gland dose, the chamber was positioned on top of the phantom’s neck region and covered with 3 mm of build-up material of silicone components which is described elsewhere [9]. The idea was to position the chamber at a depth similar to that of the thyroid gland. The setup serves as an alternative to drilling into the phantom. To obtain the breast dose, the ion chamber was positioned in a chamber-sized hole in the lateral third of a left silicone breast fitted to the phantom (Figure 1). The breast was cast from a 3D-printed mold in a procedure comparable to the production of silicone objects as described by Pollmann et al. with a volume of approximately 350 cm^3^ [9]. Finally, for the abdominal dose, the chamber was inserted into a chamber-sized hole drilled horizontally into the solid material, such that the detector was placed centrally in the phantom at navel height.

Eye lenses were protected by a disposable 0.25 mm lead-free barium-sulfate shield (SFXray, Belfast, Great Britain). A full-size 0.35 mm lead body shield (Vector X, Medical Index, Bad Rappenau, Germany) was used for protection of the thyroid gland and the body trunk. All measurements were acquired three times as described above and three times with additional shielding.

To convert the measured charge into dose, the Farmer chamber 30013 was cross-calibrated against a Semiflex 31010 ion chamber using the XVI X-ray imaging unit of a VersaHD linear accelerator (Elekta, Crawley, UK) in fluoroscopic mode (120 kV) at a fixed gantry angle. Both chambers were positioned iso-centrically and sequentially irradiated with a 10 cm circular field in a water-equivalent slab phantom at 5 cm depth. This setup replicated the calibration conditions. The Semiflex 31010 chamber had previously been calibrated for X-ray energies at similar conditions. For the final conversion of measured charge to dose, the factor for the Farmer chamber 30013 obtained from cross-calibration, an established factor accounting for energy dependence [10], as well as a compensation factor for deriving air density from temperature and pressure were applied.

## 3. Results

Normally distributed metric data are presented as means and standard deviations from repeated measurements. For CBCT studies of the wrist, the organ receiving the highest absorbed dose was the eye lens (0.10 ± 0.01 mGy). The most extensive dose reduction by means of shielding was achieved for the thyroid gland (reduction of 17%). While the highest absorbed dose in elbow studies was also determined for the eye lens (0.12 ± 0.01 mGy), a substantially higher relative dose reduction was ascertained for the ipsilateral breast tissue (39%) and, most notably, for the abdomen (48%). Absorbed dose values with and without shielding as well as the relative dose reduction are presented in Table 2.

Uncertainties of the absolute dose values are estimated as 4% (k = 2). The main contribution to the uncertainty is the calibration procedure both by the calibration laboratory and during the cross calibration. The correction of changes in the detector response due to the changed spectrum at the different measurement positions compared to the calibration conditions within the direct kV beam is not included in the dose estimate nor in the uncertainty budget.

## 4. Discussion

This experimental phantom study employed absolute dosimetry in various body regions to assess the value of added patient shielding in ultra-high-resolution extremity cone-beam CT with a gantry-free twin robotic X-ray system. Due to its tableside scan trajectory and pulsed image acquisition, the system in use allows for a relatively low dose exposure compared with multislice CT. Dedicated shield protection was observed to be more beneficial for elbow than wrist cone-beam CT in the present study.

Despite the increasing use in clinical routine, data regarding dose exposure in extremity CBCT is still limited. Some guidelines even state that specific recommendations are not feasible due to insufficient data [8]. Particularly for robotic systems without a conventional gantry, studies on the effectiveness of radiation protection efforts are lacking.

In the present investigation, the dose reduction via shield protection was substantially higher for UHR-CBCT of the elbow than of the wrist. While this result was somewhat expected given the elbow’s closer proximity to the radiation-sensitive body trunk, the marked difference in dose reduction potential between the two anatomical regions can be considered a noteworthy finding that may influence clinical routine imaging. Especially for wrist scans, patient shielding cannot be recommended based on our results, since the dose reduction for all four measurement sites was below 20%. In contrast, the effect of shielding in elbow CBCT was between 18% and 48%. It must be acknowledged, however, that the absolute radiation exposure in UHR-CBCT is generally low compared to conventional gantry-based MSCT, especially with the use of dedicated low-dose protocols, which allow for about 75% less radiation burden. Additional analyses revealed that the positions of the phantom and X-ray apron have considerable influence on the actual dose reduction, since especially the breast tissue measurement is affected by the precise positioning of the arm opening. Irrespective of the anatomical scan region, the assessment of radiation risk follows a “linear no-threshold” model, which suggests that the probability for radiation damage and radiation-induced carcinogenesis does not adhere to a minimum threshold, but decreases linearly with reduced dose exposure [11,12,13,14].

Particularly, the use of patient shielding with leaden protectors during CT scans has been a long-debated practice in medical imaging. Recent recommendations have aimed to address the effectiveness of employing lead protection for particular organs in MSCT, for example, covering the thyroid gland during head scans [8,15,16]. This guideline highlights the evolving perspective within the medical community regarding radiation protection. Traditionally, pro-shielding arguments are rooted in the intention to reduce the radiation exposure of sensitive organs and tissues outside the scanned area [8]. However, emerging evidence has challenged the effectiveness of lead protection with some studies even suggesting that lead shields may interfere with the accuracy of diagnostic imaging by introducing streak artifacts or obstructing visibility, particularly in MSCT scans [17]. While this concern is not applicable in extremity CBCT, the application of lead protection still entails certain drawbacks and inconveniences, including increased procedure time due to shield placement and patient discomfort. Considering the rather long scan time of CBCT studies (14 s), motion artifacts may be a potential consequence of uncomfortable positioning. Other factors, such as a higher degree of psychological stress and irrational concerns regarding radiation exposure may further compromise the quality and accuracy of diagnostic imaging [18]. In addition, using shield protectors for multiple patients also poses a hygiene risk, necessitating repeated time-consuming cleaning efforts [19].

Newer guidelines and recommendations emphasize the importance of evidence-based practices for radiation protection efforts. While they acknowledge scenarios where shielding might be beneficial, healthcare providers are challenged to weigh the potential benefits against the risks and limitations associated with the application of lead protection, considering individual patient characteristics, specific imaging protocols, and technological advancements in radiation dose reduction [20]. In conventional radiography, the literature analyses indicate that the dose reduction via out-of-plane shielding is relatively small in absolute terms across all applications. Given the significance of absolute organ doses for stochastic effects like carcinogenesis, dose reduction within the sub-milligray range may actually be considered insignificant [8]. While our results indicate a substantial relative effect of shield protection, particularly for the ipsilateral breast (39%) and abdomen (48%) in elbow CBCT, the absolute dose saving in all measurement sites remained marginal. Our dose savings are generally in line with the existing literature on shielding in multidetector CT: Yu et al. report that dose reduction outside the scan range resulting from use of a lead apron was 4.3–19.1% when the apron was placed at distances of 1–10 cm from the bottom of the scan range [21]. Meanwhile, Wang et al. state that the dose to the breast region was decreased by about 21% with a pediatric shield and by about 37% with adult shields [22]. Finally, İçöz et al. found that dose reduction with bismuth shielding ranged from 26.81% to 52.95% [23].

To the authors’ best knowledge, this investigation marks the first dosimetry study for extremity CBCT with a twin robotic X-ray system. Our findings provide valuable evidence on the dose burden of wrist and elbow CBCT examinations, which have become increasingly popular in recent years after the emergence of dedicated extremity scanners. Omission of shield protection in clinical care due to low absolute dose saving potential may aid diagnostic workflows and prevent unnecessary motion artifacts due to patient discomfort. Future studies should include patient-specific risk assessment as well as other anatomical regions, such as the lower extremity joints and the temporal bone region. Since multipurpose scanners capable of fluoroscopy and 3D imaging allow for a unique diagnostic application in the form of CBCT arthrography, this form of joint examination should also be investigated in upcoming research.

The presented study has certain limitations. First, the model’s abilities to simulate scatter radiation from human tissue are limited. Second, results may be reliant on the scanner architecture, limiting transferability to other systems and vendors. Third, we observed a substantial dependency on the selected examination protocol, suggesting that the dose reduction effect may be even smaller when performing examinations with a dedicated low-dose protocol. Fourth, the potential for radiation protection varies depending on the shielding measures employed. Fifth, scans were only performed with protocols used in the preoperative work-up of trauma patients. Scan settings optimized for postoperative imaging (i.e., protocols relying on a higher tube voltage) were not tested in this investigation. Finally, various approaches can be used to estimate the dose of CT examinations, such as Monte Carlo simulations and thermoluminescence dosimetry. Since this study was based solely on ion chamber measurements, our results could not be compared to these alternative methods.

Using a gantry-free scanner in combination with a tableside scan trajectory, the radiation exposure of radiosensitive organs is generally low in extremity CBCT. Although shield protection proved to be more effective in elbow than wrist scans, reducing the radiation dose by almost one third, the overall impact in terms of absolute dose reduction was marginal.

## Figures and Tables

**Table 1:** Acquisition parameters.

	Wrist and Elbow Cone-Beam CT
Tube voltage (kV)	78.3
Tube current-time product (mAs)	2.5 per pulse
Number of projections	318
Sweep angle (degrees)	200
Scan duration (s)	14
Dose-area product (cGy * cm^2^)	543.0

* Calculated for a 16 cm PMMA phantom.

**Table 2:** Absorbed dose reduction. Dose values are reported as mean ± standard deviation (in mGy). Results take into account the repeated measurements with and without patient shielding.

Dose Values in mGy	Wrist			Elbow		
	Standard	With Shielding	Dose Reduction	Standard	With Shielding	Dose Reduction
Eye lens	0.10 ± 0.01	0.09 ± 0.01	12%	0.12 ± 0.01	0.10 ± 0.00	18%
Thyroid gland	0.06 ± 0.00	0.05 ± 0.00	17%	0.08 ± 0.00	0.06 ± 0.00	21%
Breast tissue	0.09 ± 0.00	0.08 ± 0.00	13%	0.10 ± 0.00	0.06 ± 0.00	39%
Abdomen	0.07 ± 0.00	0.07 ± 0.00	10%	0.07 ± 0.00	0.03 ± 0.00	48%

## Data Availability

The original contributions presented in this study are included in the article. Further inquiries can be directed to the corresponding author. Multitom Rax VF11 with 3D function is not available in all countries. Its future availability cannot be guaranteed.
